# Fast DLLME-GC-MS Method for Determination of Pesticides in Carmelite Drops and Evaluation of Matrix Effects in Related Medicinal Products

**DOI:** 10.3390/foods13111745

**Published:** 2024-06-02

**Authors:** Agneša Szarka, Svetlana Hrouzková

**Affiliations:** Institute of Analytical Chemistry, Faculty of Chemical and Food Technology, Slovak University of Technology, Radlinského 9, 812 37 Bratislava, Slovakia; agnesa.szarka@stuba.sk

**Keywords:** matrix effects, matrix factor, DLLME-GC-MS, Carmelite drops

## Abstract

The production of nutraceuticals is a growing trend, as many consumers consider them an important part of the modern active lifestyle. Others rely on the use of nutraceuticals instead of prescribing pharmaceuticals to improve their health more naturally. One of the major concerns in the nutraceutical industry is the potential presence of contaminants. Even low concentrations of contaminant residues can be harmful, so analytical methods that are sensitive at ultratrace levels are needed. Dispersive liquid–liquid microextraction method combined with fast gas chromatography and mass spectrometry was developed for the inspection of pesticide residues in Carmelite drops. The most suitable recoveries are presented when the alcohol content is fixed at 20% in Carmelite drops. The method was validated; the linearity, limits of detection/quantification, the method accuracy and precision were obtained. The complex nutraceutical matrix causes significant complications in quantitative analysis; therefore, the main target of the work was placed on studying the effects of the matrix on the correct expression of the resulting concentration of contaminants in different types of samples. An in-depth study of matrix factors was carried out, and its relationship with the content of potential interferents from the medicinal products as well as other components added during the drops’ production was discussed. Related medicinal plant-derived nutraceuticals were tested, the method was applied for real-life samples, and positive findings are herein reported.

## 1. Introduction

Herbal medicines have been utilized for a variety of purposes, including dietary ingredients and therapies, since ancient times. Even though synthetic medications help treat a variety of disorders in the modern period, there is growing interest in compounds that have the therapeutic benefits of plants that grow in the wild [1]. These plants have medical potential because of a few chemically active ingredients that have specific physiological effects on humans. Among these chemically active plant components, alkaloids, tannins, flavonoids, and phenolic chemicals are the most significant. Many of these naturally occurring medicinal plants are also employed in medicine [2]. Functional foods can help reduce the risk of certain diseases and other health-related disorders [3]. Researchers are closely examining antimicrobial compounds in these products due to the acceptance of traditional medicine as an alternative type of healthcare [4]. Antimicrobials of medicinal plant extracts are natural, safer than synthetic alternatives, available in local communities, cheaper to purchase, easy to administer, and can offer profound therapeutic benefits and more affordable treatment [5,6,7]. Plants and herbs can be prepared and utilized in a variety of ways. These include whole herbs, teas, syrups, tinctures, essential oils, ointments, liniments, and capsules and tablets that contain a pulverized or powdered form of a raw herb or plant or its dried extract [8]. Alcohol and water extracts of plant materials are typically used to make tinctures. In a combination of alcohol and water, many plant elements dissolve more readily than in pure water [9]. Several structurally varied chemicals with various polarities are extracted during the maceration of plant components in water–ethanol solutions used to prepare tinctures. Quality-control analytical instruments that are tailored for the identification of individual chemical compounds or a particular group of compounds are necessary due to the great chemical variety of the contents [8].

Plant products are particularly susceptible to contamination at almost every stage of production, including growth conditions, open-air drying, preservation, manufacturing, and storage. Most botanicals or herbals used as the basis for dietary supplements are grown using traditional agricultural methods, which may include the use of pesticides [10]. The competent authorities, such as the Japan Food Chemical Research Foundation (Japan), the Environmental Protection Agency (United States), EFSA (Europe), FSSAI and Ayush Department (India), and others, publish pesticide residue limits for food commodities including botanical products. For commodities and insecticides for which a limit is not specified, the maximum residue limit by default is used. The default maximum residue limit (MRL) of 10 µg/kg is implemented in the U.S. and the EU following 40 CFR 180 [11] and EU regulation 396/2005 [12]. Nutraceutical products are not currently covered by legislation, but because of possible manufacturing process contamination, both raw materials and other by-products can become contaminated. Furthermore, several investigations have revealed the exclusive existence of pesticides [13,14,15] in several dietary supplements, underscoring the need to assess the contamination profile of these commodities in light of their growing popularity and use. Uncontrolled pesticide use has negative effects and puts consumers at significant risk when it comes to commodities imported from underdeveloped countries [16].

Compared to other food products, there are fewer techniques available for the extraction and analysis of pesticides from plant-derived nutraceuticals [11]. Nutraceutical products have a very complex matrix in addition to being mostly concentrated plant forms, which means that large quantities of pigments, lipids, and other co-extractives are present [16,17,18]. A suitable sample preparation method and clean-up are necessary to separate and concentrate the target chemicals to detect pesticide residues. Due to the labor-intensive and time-consuming nature, low enrichment factors (EFs) of most pretreatment techniques used for these kinds of samples, and the need for large quantities of hazardous solvents, various sorbent- and solvent-based microextraction techniques have gained popularity recently. For the examination of pesticides in nutraceutical drops, dispersive liquid–liquid microextraction (DLLME) should be an appropriate extraction technique. It is necessary to use an additional organic disperser solvent in DLLME, which often lowers the hydrophobic analytes’ partition coefficients into the extraction solvent [19,20]. Consequently, alcohol-containing samples, like nutraceutical drops, might be of interest to DLLME. In this context, the extraction step’s dispersive solvent was swapped out for ethanol [19,21]. Like several pesticides, co-extracted matrix ingredients show both partitioning behavior and chromatographic features, which can cause major interference and harmful matrix effects (MEs) in mass spectrometry analysis.

ME is considered a major problem in gas chromatography (GC-MS) and liquid chromatography–mass spectrometry or tandem mass spectrometry (LC-MS, LC-MS/MS) quantitative analysis that has been addressed with attempts to overcome it in several articles. ME is defined by the International Union of Pure and Applied Chemistry (IUPAC) as “the combined effect of all components of the sample other than the analyte on the measurement of the quantity” [3]. Due to competition between the analytes and non-volatile matrix components (fatty acids, glycerates, salts, etc.), which reduces the analyte ion production efficiency, the former typically indicates inhibited MEs [22]. The latter typically presents enhanced MEs because of matrix components that accelerate the mass transfer of the target analytes by blocking active sites in the capillary column or the injector liner [23]. However, our understanding of the exact mechanisms behind MEs is still incomplete [24]. Using a normal standard calibration curve to determine the residue levels will lead to erroneous quantification conclusions if the matrix impact is considerable in the analysis. The accuracy of quantitative analysis can be improved by using internal standards (IS) in multiresidue analysis. The retention period of IS should be the same as the analytes or rather similar. Furthermore, the co-eluted matrix ought to have an equal impact on both compounds [25]. It is possible to apply a stable isotopically labeled internal standard (SIL-IS) or a structural analog as IS [26]. SIL-IS compounds are those in which stable isotopes of various analyte atoms, such as 2H (deuterium), 13C, 15N, or 17O, are substituted for some of the atoms [27]. As a result, SIL-IS has been deemed the best alternative to structural IS analogs and is ideally suited for these uses. To prevent an unanticipated behavior between the analyte and the SIL-IS, the existence of MES should be assessed in any instance, even if an SIL-IS is used [28]. Regrettably, this methodology also has several drawbacks, like the low availability and expensive nature of SIL-IS. One major disadvantage is that applying a wide range of IS to a multi-component analytical situation can be challenging. In many cases, it may be extremely difficult or impossible to find an appropriate IS for every analyte [25]. Nowadays, matrix-matched calibration is the technique that analysts utilize most frequently to reduce matrix effects. However, because they only serve as corrective measures rather than purifying contaminants that produce MEs, calibration techniques based on IS or matrix-matched calibration cannot be utilized to correct near limit of quantification (LOQ) values [29,30]. Therefore, increasing clean-up efficiency is the most effective strategy to lessen the effects of the matrix [31]. Nevertheless, proper clean-up optimization is required because the target analytes could also be lost during the purification processes, so it is advised to investigate the recoveries.

The current study aimed to develop a fast GC-MS method for pesticide residues determination in Carmelite drops, which are complex medicinal nutraceutical drops. Microextraction DLLME was used for sample preparation. For the research, 40 individual pesticides were investigated. The developed method was validated for Carmelite drops. Additionally, six different types of related medicinal products were selected to test matrix effects. The work focuses on the study of the effect of alcohol content in nutraceutical drops on extraction recovery.

## 2. Materials and Methods

### 2.1. Chemicals and Reagents

Different sources (Dr. Ehrenstorfer, Augsburg, Germany; Bayer, Leverkusen, Germany, Chromservis SK, Bratislava, Slovakia) provided high-purity standards of pesticides from various chemical groups (organophosphorus, organochlorine, chloroacetamide, triazole, pyrethroid, dicarboximide, pyrimidine, phenols, amines, oxazoles, and carbamate pesticides), the details of which are listed in Table 1. The selection of the pesticides was based on several categories: (1) multi-residual character; (2) pesticides that could be expected in herbal samples; and (3) pesticides that are suspected to be endocrine-disrupting chemicals.

One milligram per milliliter of the pesticide standards stock solution was made in ethanol (Suprasolv grade, Merck KGaA, Darmstadt, Germany). A composite solution containing 0.02 mg/mL of all pesticides was prepared. The following solvents, tetrachloroethane (manipulation in digester by using proper eye and skin protection to prevent acute dermal and inhalation toxicity), and ultra-pure water (Sigma Aldrich, Steinheim, Germany) were used for the DLLME procedure. Preferably, reagent-grade or pesticide residue-grade-purity solvents were utilized. By appropriately diluting a mixture, the working pesticide solutions were created. The diluted working solutions were held at +4 °C, while stock solutions were kept frozen at −18 °C.

### 2.2. Instrumental Equipment and Conditions

An Agilent 6890N gas chromatograph (Agilent Technologies, Little Falls, DE, USA) hyphenated with an Agilent 5975 mass-selective detector was used for chromatographic studies. A programmable temperature vaporization injector (PTV) and an Agilent 7683B autosampler were utilized to inject 2 μL of solutions in solvent vent injection mode. Initial settings for the PTV injector temperature were 80 °C (hold 0.20 min), followed by 300 °C (400 °C/min, hold 2.00 min), and finally 350 °C (400 °C/min, hold 5.00 min). A non-polar deactivated pre-column (1 m × 0.32 mm I.D.) was linked to a CP-Sil 8 CB (Agilent Technologies, Middelburg, The Netherlands) gas chromatographic column with a chemically bonded 5% diphenyl 95% dimethylsiloxane stationary phase, with dimensions 15 m × 0.15 mm I.D. × 0.15 μm film thickness. The column temperature was first set at 100 °C and held there for 1.75 min. It was then gradually increased to 150 °C at a rate of 60 °C/min and finally to 300 °C at a rate of 23.8 °C/min. The last isothermal phase was held for 2.90 min. The carrier gas was helium, flowing at a constant 1.2 mL/min rate.

The electron ionization mode (70 eV) was utilized when operating the mass spectrometer. The temperature of the quadrupole and the ionization source were set at 150 °C and 280 °C, respectively. The mass range of 40–550 *m*/*z* was chosen for the full scan. A three-minute solvent delay was used in MS.

Sartorius Analytic MC1 scales (Sartorius, Göttingen, Germany) were used to weigh pesticides. The sample was prepared using the ROTOFIX 32 Hettich centrifuge (Tuttlingen, Germany) and Vortex HEIDOLPH (Multireax, Schwabach, Germany).

### 2.3. Samples and Sample Preparation

Medicinal nutraceutical Carmelite drops (previously checked for the presence of the target pesticide residues) were purchased from a bio store in Bratislava, Slovakia, and utilized as a blank matrix to create fortified samples for the recovery tests and matrix-matched reference solutions for the calibration and the matrix effects studies. Carmelite drops were kept at 5 °C until the time of analysis.

For the DLLME technique, a 15 mL centrifuge tube was filled with 1 mL of a fortified nutraceutical drop containing 40% ethanol. The fortified sample was mixed with 0.01 g of NaCl and 1.75 mL of ultra-pure water. Then, a rapid injection was performed using a combination of 80 µL of tetrachloroethane (the extraction solvent) and 187.5 µL of methanol (the dispersive solvent). The dispersion of the tiny tetrachloroethane droplets in the aqueous–alcoholic solution created a cloudy system in the centrifuge tube. The closed centrifuge probe was rapidly shaken by hand, vortexed for three minutes at 1800 rpm, and then centrifuged for two minutes at 4000 rpm. The final extract that settled at the centrifuge probe’s bottom was analyzed by GC-MS method.

The sample of Carmelite drops used in the investigation of the effect of alcohol content on extraction recovery had an initial alcohol content of 40%. This was varied by adding ethanol or water to the samples to adjust the alcohol percentage between 10% and 60%, and the effect of alcohol concentration on the DLLME extraction efficiency of forty pesticides was investigated. These samples were subjected to a recovery test at a spiking concentration of 50 μg/L.

### 2.4. Matrix Effects

Each pesticide’s matrix factor (MF) was determined to recognize the ME. The following formula was used to determine the MFs for each pesticide under investigation by comparing the analyte response in a matrix-matched solution with the pesticide response obtained in pure solvent at a concentration level of 50 μg/L.

(1) $$MF=\left(\frac{\begin{array}{cccc} response & in & matrix-matched & solution \end{array}}{\begin{array}{cccc} response & in & solvent & solution \end{array}}-1\right)\begin{array}{cc} \times & 100\% \end{array}$$

For the calculation of MFs, peak areas for quantification ion in SIM mode of GC-MS analysis of each pesticide (shown in bold in Table 1) were utilized. The order of the injection in the sequences for the study of matrix effects was as follows: (1) pure solvent solution of the analytes and (2) matrix-matched solution (Carmelite drops) with the following three repetitions. In the same way, the sequences of grapefruit, milk thistle, echinacea, capsella, and willow drops followed. Samples showing the absence of the target compounds were used to prepare matrix-matched standard solutions for the calculation of the MF.

### 2.5. Validation Process

Using matrix-matched standard solutions at ten different concentration levels (0.01, 0.05, 0.1, 0.5, 1, 5, 10, 50, 100, and 250 µg/L), linearity was assessed. Relative peak area was used as the analytical signal for a linear least square regression analysis. The calibration curve featured a zero point to ensure that the blank samples were free of pesticides. The lowest calibration level (LCL) was selected individually depending on the pesticide response. Each spiked extract was analyzed in triplicate.

For recovery studies, samples were spiked at three different concentrations (10, 50, and 100 µg/L), and five replicates were used for each level. Blank soya-based nutraceuticals were fortified with pesticides before extraction. The spiked samples were left to stand for 30 min before their extraction. The recovery was used to calculate accuracy, which was performed in five repetitions at three spiking levels of 10, 50, and 250 µg/LF. The analyte amount in the sediment phase (*n*_sed_) divided by the total amount of analyte (*n*_0_) was used to express recoveries, and this ratio was typically stated as a percentage:

(2) $$ER=\frac{n_{\mathrm{sed}}}{n_{0}}\times 100\%=\frac{c_{\mathrm{sed}}\times V_{\mathrm{sed}}}{c_{0}\times V_{\mathrm{aq}}}\times 100\%$$

where *V*_sed_ and *V*_aq_ stand for the relative volumes of the sediment phase and sample solution. The initial analyte concentration in the sample is denoted by *c*_0_, while the analyte concentration in the sedimented phase is represented by *c*_sed_. The precision was expressed as intra-day and inter-day precision by the relative standard deviation (RSD).

The estimation of LOQs and limits of detection (LODs) was the last step. The concentration corresponding to the levels of signal-to-noise ratios of 3:1 and 10:1, respectively, was considered LOQ and LOD. The proper LCL was used for signal-to-noise ratio evaluation.

### 2.6. Real Samples

During the analysis of samples, 10 different nutraceutical drops were obtained from local supermarkets (Bratislava, Slovakia). Several drops were stored at 5 °C until the moment of analysis. The alcohol content of the real samples was in the range of 24.5–70%, and they contained different types of herbs. Five samples of nutraceuticals containing 40% alcohol were chosen, and they were prepared following the directions given in Section 2.3. The alcohol percentage of the remaining five samples ranged from 24.5% to 70%. Before the studies, the alcohol content had to be adjusted to 40% using either ethanol or water, depending on the sample.

## 3. Results and Discussion

This study investigated forty pesticides that belonged to diverse chemical classes, including carbamates, amines, phenols, dinitroanilines, organochlorines, azoles, pyrimidines, and pyrethroids, which have varying physical and chemical properties. At first, the working solution of standards prepared in the neat solvent of tetrachloroethane at a concentration of 1 ng/µL was analyzed in FS mode, and then, the pesticides were initially categorized into selected ion monitoring (SIM) groups based on their retention times. The examined pesticides, polarity indicated by K_ow_, retention times, and monitored *m*/*z* fragment ions are all included in Table 1.

### 3.1. The Effect of Alcohol Content on DLLME Efficiency

A concentrated liquid form of one or more herbs is an herbal tincture. The active ingredients in the herb or herbs are extracted during the soaking procedure. Since alcohol may extract non-water-soluble components like alkaloids and resins, it is frequently the liquid of choice. Traditional and folk medicines frequently contain a high alcohol content, and the extraction of bioactive components from plants may require the use of alcohol [33]. An additional benefit of alcohol in tinctures is that it works as a preservative, extending the shelf life of the extract for several years. To properly preserve a completed extract, its alcohol level must be at least 20% *v*/*v* [8]. The majority of tinctures made for commercial use have a minimum alcohol level of 25% *v*/*v*. Optimizing the quality of herbal remedies requires using the appropriate ethanol concentration. [8].

In this study, nutraceutical drops of plant origin with mostly 40% ethanol content were applied. In the DLLME process, a dispersing solvent is typically used to encourage the extractant into microdroplets. However, in the DLLME process, ethanol in nutraceutical drops could be utilized as the disperser solvent [19,21,34,35], and no other disperser solvent was added [21].

**Figure 1:**
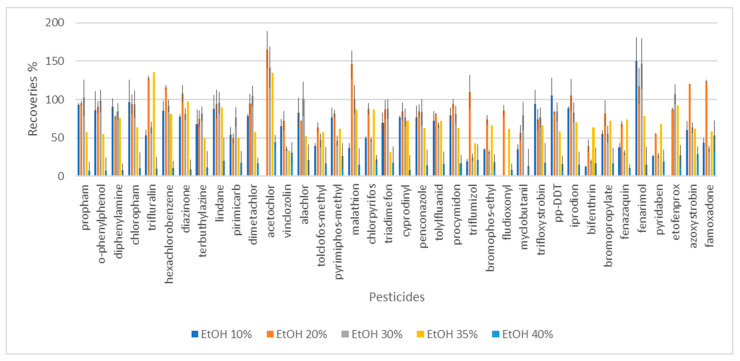
The recoveries of pesticides in the dependence on the ethanol content in Carmelite drop sample (DLLME of 4 mL sample with 80 µL of tetrachloroethane).

At first, the dispersion and the sedimentation of the extract were studied during the extraction for samples with ethanol contents of 10%, 20%, 30%, 40%, 50%, and 60%. (The ethanol content in Carmelite drops was adjusted by dilution or by addition of ethanol into the sample.) Rarely can microdroplets form when a small amount of disperser solvent is utilized. Nevertheless, a high disperser solvent content will hinder the organic phases from separating [36]. No separation of the extract and the sample phase was observed for samples with ethanol content higher than 40%. Higher volumes of the extractive solvent (100 and 120 µL instead of 80 µL) were tested to improve the separation, but it was not successful. Therefore, the recoveries of pesticides were studied for the DLLME of nutraceutical drops containing alcohol at a range of 10–40%. The dependence of the recoveries of individual pesticides on different alcohol content is shown in Figure 1.

It should be seen that with the growing content of ethanol, the recoveries for most of the pesticides increased, and at the range of 20–30% there were no significant differences. The optimal recoveries for pesticide residue analysis were set at the range of 70–120% according to the SANTE [37] document. Most of the pesticides with recoveries in the given range were extracted when the alcohol content in the sample was 20%. Overall, 28 pesticides presented recoveries of 72 and 120%, and 4 other pesticides, namely trifluralin (128%), tolclofos-methyl (64%), fenazaquin (68%), and famoxadone (124%), were near to the optimal range. The number of pesticides with appropriate recoveries decreased with the higher content of ethanol in the sample; for 30% ethanol content, 21 pesticides showed recoveries in the range of 70–120%; for 35%, 13 pesticides; and for 40% ethanol content, none of the pesticides were extracted with recoveries at the optimal range. The relatively high volume of ethanol increases the solubility of target analytes in the aqueous phase and, as a result, reduces the partition coefficients of analytes into the extraction solvent [19,20].

The recoveries were performed on five replicates, and the precision was expressed by the relative standard deviation (RSD). RSD values were lower than 20% for all analytes when the ethanol content was 20%, fulfilling the established requirements for pesticide residue analysis. However, the ethanol content was higher than 20% some of the RSDs were out of the suitable range:

- Alcohol content 10%, RSD in range 2–30%;
- Alcohol content 30%, RSD range 6–23%;
- Alcohol content 40%, RSD range 5–30%.

The recoveries of pesticides were statistically evaluated (ANOVA test). There was no significant difference between the data obtained for the samples with 20% and 30% of alcohol. this means that for plant-derived nutraceutical drops with an alcohol content of 20% or 30%, the DLLME extraction procedure should be used without the modification of the alcohol content. Upon correlation of the data obtained for other samples with alcohol contents of 10, 35, and 40%, a significant difference was obtained, which means that correction of the ethanol percentage (to 20%, which presented the best extraction recoveries and RSDs) is needed before the extraction process. In the case of significant effects, the Bonferroni post hoc test was conducted. According to the Bonferroni post hoc test, we may infer that the statistically significant difference in mean was discovered between sample recoveries with alcohol contents of 10 and 20%, 10 and 40%, 20 and 40%, and 30 and 40%. According to the Kolmogorov–Smirnov test, the recoveries of pesticides were not normally distributed in samples with different alcohol contents, except for the sample comprised of 20% alcohol.

**Figure 2:**
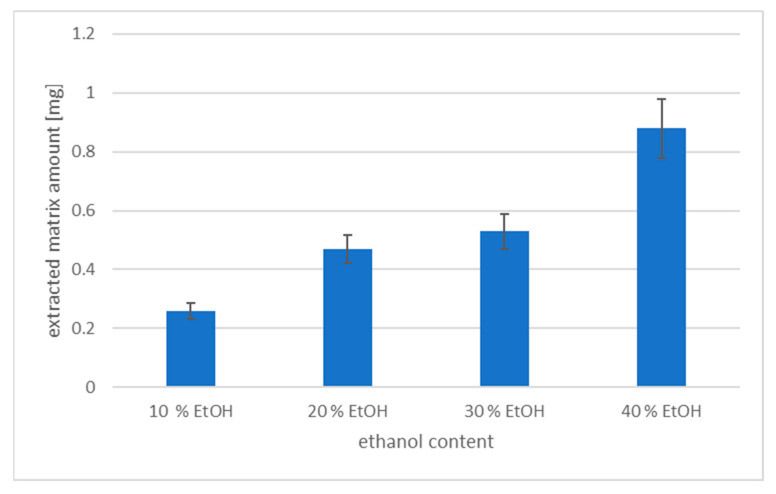
The amount of the extracted matrix in mg from samples with different alcohol content (DLLME, 4 mL sample, 80 µL of tetrachloroethane).

For the comparison of the extracted matrix interferences during the DLLME, the final extracts of the samples with different alcohol contents (10, 20, 30, and 40%) were evaporated until dryness under the stream of nitrogen, and the dry matter was weighted. It was obtained that the percentage of alcohol in the sample affected the extracted amount of the matrix. The weight of the dry matter was increased with the amount of ethanol in the sample. The ethanol improved the ability of the extraction of the matrix impurities, which should have a negative impact on method sensitivity. It corresponds with the recovery studies, which show that the higher content of ethanol decreases the extraction recovery. In Figure 2, the dependence of the dried matter amount (in mg) on the ethanol content of the sample is depicted.

The most suitable recoveries were obtained when the alcohol content in medical plant-derived nutraceutical drops was adjusted to 20%; therefore, for the study of the matrix effects and validation experiments, the ethanol content in all the kinds of samples was adjusted to 20% before the DLLME.

### 3.2. The Evaluation of the MEs in Related Medicinal Drops

Improved mass transfer of the target analyte can be achieved in GC-MS analysis by blocking the active sites in the capillary column or the injector liner with matrix components. As a result, one of the main elements influencing pesticide MEs is the matrix. Because they contain a higher concentration of co-extractives and unique active ingredients such as sugars, phenolics, flavonoids, natural pigments, and essential oils, medicinal nutraceutical drops have more substantial MEs. MEs in six representative medicinal nutraceutical drops were investigated in this study, which is presented in Table 2. A tincture is a plant substance’s active ingredients extracted liquidly using a water and alcohol solution. A tincture’s alcohol content varies according to what works best for a given botanical or plant component. Botanical tinctures have an approximate alcohol level of 20–90%. Generally, less alcohol is required when using the plant’s aerial parts (leaves, stems, and flowers). Samples with alcohol contents ranging from 24.5 to 66% were examined in this study. The volume of the samples was 50 mL.

**Figure 3:**
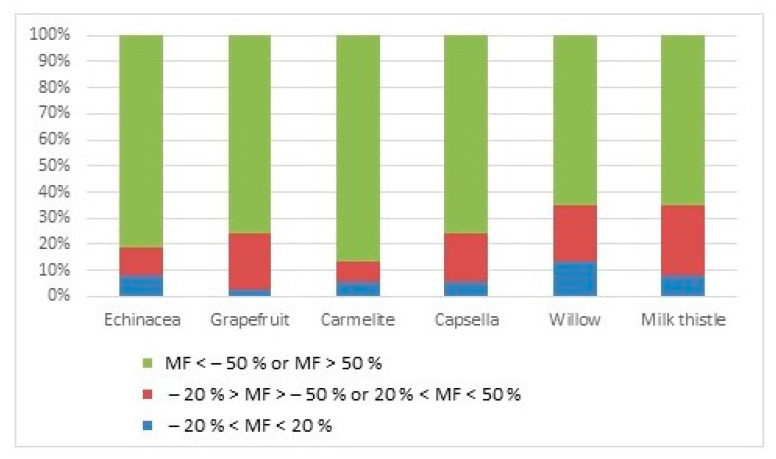
The percentage of pesticides showed minimal, moderate, or strong ME in the studied matrices (DLLME, 4 mL sample ethanol content adjusted to 20%, 80 µL of tetrachloroethane).

In the study, both signal suppression and signal enhancement were determined depending on the analyte and matrix combination. MFs higher than 20% or less than −20% indicate an increased or suppressed peak signal, respectively. MEs were classified into three types: minimal signal suppression or enhancement effects (MF interval −20% to 20%), moderate effects (MF interval −50% to −20% or 20% to 50%), and strong MEs (less than −50% or greater than 50%) [19,38]. Figure 3 indicates that most of the pesticides are influenced by strong MEs (MF > 50%) in all tested matrices.

In GC analysis, signal suppression is less common; in some matrices, only 16% of the pesticides examined exhibited signal suppression. A small number of chemicals showed the reported signal suppression, including terbuthylazine, fludioxonil, and triadimefon. The signal of these pesticides was suppressed by the matrix in three or more types of samples. Most of the pesticides affected by the minimal ME were in willow drop samples. The sum of the pesticides with minimal and moderate MEs was similar for willow and milk thistle drops. Thirty-five percent of the studied pesticides showed minimal or moderate ME in these two samples, which means that these two samples provide a cleaner sample extract in comparison with the other four samples. The highest number of pesticides with MF higher than 50% were observed for Carmelite drops (86% of pesticides). Carmelite drops include the extract of different types of herbs, like lemon balm leaves, lemon perch, nutmeg, cinnamon bark, and clove flower; therefore, the matrix of the sample is more complex than for nutraceutical drops, which are made from one type of the herb; thus, the extract is more loaded with interferences. DLLME extracts of Carmelite drops were rich in pigment compared to other samples. Therefore, pigments in matrices may be one of the factors that lead to MEs [24]. However, this is still a hypothesis, and more research is required to validate it.

Since co-extractives were separated within the same chromatographic run, it was revealed that the elution conditions had a significant impact on the MEs of pesticides [24]. As a result, this study also covered the impact of the analytes’ retention time. From the obtained results, it should be seen that the MFs in all types of the samples increased with the retention times of the pesticides (Table 3). Therefore, it can be expected that the physicochemical properties of some coextracted matrix substituents were similar to those of pesticides eluted in higher retention times windows.

**Figure 4:**
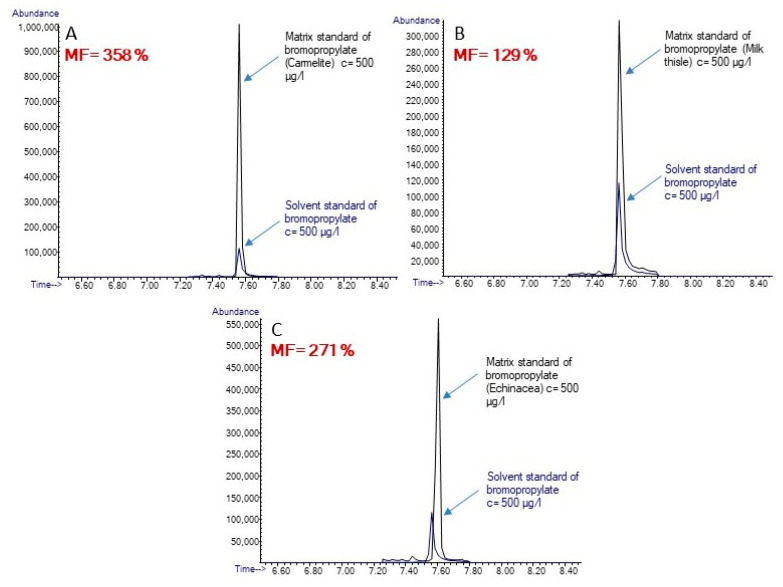
Extracted ion chromatograms for bromopropylate in solvent and as a matrix standard in Carmelite drops (**A**), milk thistle drops (**B**), and echinacea drops (**C**).

Table 3 summarizes the MFs for all studied pesticides in six different samples. The ME was studied in three replicates, and the reproducibility was expressed by RSD. The RSD for all of the samples was in the range from 0.5 to 15%. Some of the pesticides were affected by a very strong ME, namely lindane (in echinacea drops), cyprodinil (in echinacea drops), trifloxystrobin (in echinacea and grapefruit drops), fenazaquin (in grapefruit, capsella, and willow drops), and azoxystrobin (in grapefruit, Carmelite, capsella, and willow drops). The MF for most of the pesticides was higher than 100%. The highest MFs were obtained in echinacea, grapefruit, and Carmelite drops, with the highest MF of 2379% for triflumizol in Carmelite drops. On the other hand, in the case of capsella, milk thistle, and willow drops, 16, 15, and 16 pesticides, respectively, presented a higher MF than 100%. For illustration, ME for bromopropylate is shown in Figure 4.

The fact that most of the MFs fall outside of the satisfactory range indicates significant disparities between the matrix-matched standards and the solvent-matched standards. These days, laboratories use matrix-matched standards, an efficient method for eliminating MEs. To facilitate method validation in pesticide analysis, the SANTE document generally advises using matrix calibration, recognizing the ME as a crucial component of method validation [37]. The results of the study confirm this statement.

### 3.3. Validation of the Method

The developed analytical method for the determination of pesticide residues in Carmelite drops was validated, which represented the most complex matrices with the highest number of pesticides with strong MEs. In the concentration range of 0.01–250 µg/L, linearity was investigated. The calibration curves obtained by studied pesticides showed a linear dynamic range for a minimum of five orders of magnitude. For the calibration curves with five to nine data points (in the dependence on LCL reached), the signal intensities used for each data point were averaged from six repeated injections. Good linearity was observed for each pesticide with coefficients of determination higher than 0.97. The LCL of the pesticides was in the range of 0.01–5 µg/L. The LCLs and correlation coefficients are displayed in Table 4.

The LODs and LOQs were calculated using the signal-to-noise ratio (S/N) values of 3 and 10, respectively, and data on LOD are summarized in Table 4. The obtained LODs fell between 0.002–0.416 µg/L, with standard error 1.10^−3^–8.10^−3^ µg/L. The lowest LODs were obtained for trifluralin (0.002–0.003 µg/L) in all types of matrices. This could be explained by the moderate MEs of the samples to the signal of trifluralin. The LODs of pesticides in echinacea drops were in the range of 0.002–0.454 µg/L, in grapefruit drops 0.002–0.283 µg/L, in Carmelite drops 0.002–0.416 µg/L, in capsella drops 0.002–2.038 µg/L, in willow drops 0.002–1.153 µg/L and in milk thistle drops 0.003–0.882 µg/L. The highest LODs were obtained for diphenylamine (2.017 µg/L) and chlorpropham (2.038 µg/L) in capsella drops and lindane (1.153 µg/L) in willow drops. The LOD of lindane was high in all types of matrices, except for grapefruit drops, in comparison with the other compounds. In most of the samples, the highest levels of LODs were obtained for this compound. The obtained LODs could not be compared with the MRLs because MRLs for nutraceutical products are not defined by the European Union, only in some of the raw herbs and herbal infusions. The MRLs defined by the European Union for most of the studied pesticides are in the range of 10–500 µg/L in herbs and herbal infusions, which is much higher than the LODs and LOQs obtained by the developed method.

**Figure 5:**
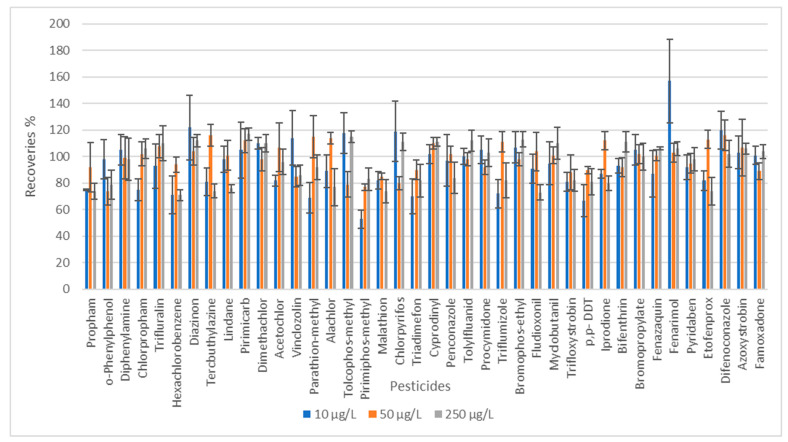
The recoveries of pesticides in three concentration levels. Sample: Carmelite drops.

The recoveries at three different concentration levels were studied in Carmelite drops samples, which represent the matrix with different types of herbs, like lemon balm leaves, lemon perch, nutmeg, cinnamon bark, and clove flower; therefore, the matrix of the sample is more complex than for nutraceutical drops. which are made from one type of herb. The blank samples of Carmelite drops were fortified to concentrations of 10, 50, and 250 µg/L. Moreover, 10 µg/L represents the default MRL for commodities for which a limit is not specified, such as nutraceutical drops, and 250 µg/L was the highest concentration of calibration solutions. The recoveries of more than 90% of pesticides were in a suitable range, between 70 and 120%. It can be seen in Figure 5 that some of the pesticides were out of the suitable range at the lowest concentration level: Fenarimol showed higher recovery than 120% (157%), and three pesticides, namely parathion methyl, pyrimiphos methyl, and p,p-DDT, showed recoveries lower than 70%.

**Figure 6:**
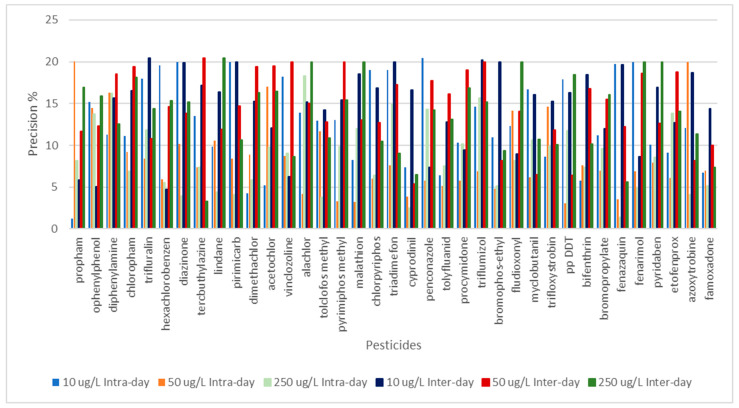
Inter-day and intra-day precision for pesticides at three concentration levels. Sample: Carmelite drops.

Five replicates were analyzed for all concentration levels, and the precision was expressed by RSD values, which were lower than 20% for all analytes (Figure 6), fulfilling the established requirements for pesticide residue analysis.

Precision was studied in terms of repeatability (inter-day precision) and intermediate precision (intra-day precision). The obtained values were expressed as RSD (see Figure 5). Intermediate precision values ranged between 1 and 20%. In the case of inter-day precision, the RSD values were lower than 20% for all pesticides at all checked concentration levels, which suggests that the analytical method is appropriate for the analysis of real-life samples.

### 3.4. Real Sample Analysis

Ten real samples with different alcohol contents were analyzed. Three pesticides, namely tolclofos-methyl, o-phenylphenol, and bromopropylate, were determined in nutraceutical samples using a DLLME extraction followed by gas chromatography–mass spectrometry analyses (Table 5). The MRLs for nutraceutical drops are not defined by the European Union (EU), although they are set in raw material, such as citrus fruits or some herbs. The detected concentrations do not exceed these limits.

Tolclofos-methyl was detected in the echinacea sample at a concentration of 2.24 µg/L. The sample is an aqueous–ethanol extract of the fresh flowering inflorescence of the *Echinacea purpurea* plant. The latter contains substances that improve human immunity. Tolclofos-methyl is a fungicide used to protect seeds against pathogenic fungi. It is used to protect various crops such as corn, soybeans, potatoes, sugar beets, and wheat [39]. Although it is not used directly for the protection of *Echinacea purpurea*, there is more potential for its contamination from other sources through soil and water. No MRL has been established for tolclofos-methyl for herbs and edible flowers.

Two pesticides, namely o-phenylphenol and bromopropylate, were detected in a sample of Carmelite drops. O-phenylphenol was present in the sample at a concentration of 16.47 µg/L and bromopropylate at a concentration of 0.67 µg/L. Carmelite drops consist of a 10% extract of a mixture of herbs (lemon balm wort, lemon balm pericarp, nutmeg, cinnamon bark of the Ceylon tree, and clove blossom of fragrant clove) in 40% alcohol. O-phenylphenol is an agricultural fungicide used after harvest. It is often used to wax citrus fruits (2-phenylphenol, 2018). Bromopropylate is a chemical compound that is used as an acaricide against mites on fruit crops such as citrus and grapes [40]. Both pesticides are used to protect citrus fruits, which include the true lemon tree, the pericarp of which is found in Carmelite drops. The MRL of o-phenylphenol in citrus fruits is 10 mg/kg, and the MRL of bromopropylate is 10 µg/L.

The positive findings endorse the idea that a deeper and continuous investigation of pesticide residues in nutraceutical products is necessary to guarantee the safety of consumers. Considering the amount of pesticides detected in the analyzed samples, we could assume that it is necessary to establish MRLs for these kinds of products.

## 4. Conclusions

To determine pesticide residue in Carmelite drops, the analytical method utilizing DLLME for sample preparation and fast GC-MS was developed and validated for Carmelite drop nutraceuticals, which represent the most complex sample, with different herbal contents. The method provided satisfactory linearity, high accuracy and good repeatability. The relationship between sample alcohol content and extraction recoveries was investigated, and it was concluded that the ethanol content needed to be adjusted to achieve adequate recovery. The matrix impact in six medicinal nutraceutical drops, namely echinacea, grapefruit, Carmelite, capsella, willow, and milk thistle, was studied. It was demonstrated that the complexity of Carmelite, capsella, grapefruit, and echinacea drops has a matrix factor, indicating high matrix effects for the majority of pesticides. Thirteen out of the thirty-seven pesticides indicated minor or moderate matrix effects in the case of willow and milk thistle drops. It may be deduced that the use of matrix-matched standards is highly recommended in the case of complex matrices such as medical nutraceutical drops.

## Figures and Tables

**Table 1:** List of pesticides, their polarity, and GC-MS parameters (retention time and monitored ions).

No.	Pesticide	log K_ow_	Retention Time (min)	Monitored Ions (*m*/*z*)
1	Propham	2.60	3.811	**179**, 93, 120
2	o-Phenylphenol	3.18	4.119	**170**, 169, 141
3	Diphenylamine	3.82	4.553	**169**, 168, 167
4	Trifluralin	5.27	4.578	**306**, 264, 307
5	Chlorpropham	3.4	4.673	**127**, 171, 213
6	Hexachlorobenzene	3.93	4.864	**284**, 286, 249
7	Terbuthylazine	3.70	5.085	**214**, 229, 173
8	Diazinon	3.69	5.103	**304**, 179, 107
9	Lindane	3.50	5.151	**181**, 219, 183
10	Pirimicarb	1.70	5.380	**166**, 72, 238
11	Acetochlor	4.14	5.420	**149**, 146, 223
12	Vinclozolin	3.02	5.545	**212**, 285, 178
13	Dimethachlor	2.17	5.552	**197**, 134, 149
14	Alachlor	3.09	5.620	**160**, 188, 146
15	Tolclofos-methyl		5.631	**265**, 267
16	Parathion-methyl	3.00	5.650	**263**, 125, 109
17	Pirimiphos-methyl	3.90	5.772	**290**, 305, 276
18	Malathion	2.75	5.790	**173**, 127, 93
19	Chlorpyrifos	4.70	5.945	**314**, 197, 199
20	Triadimefon	2.77	6.099	**208**, 181, 210
21	Cyprodinil	4.00	6.291	**224**, 225, 86
22	Tolylfluanid	3.90	6.327	**137**, 238, 101
23	Penconazole	4.64	6.356	**248**, 159, 161
24	Triflumizole	3.67	6.446	**278**, 206, 179
25	Bromophos-ethyl	6.15	6.450	**359**, 303, 242
26	Procymidone	3.30	6.450	**283**, 285, 96
27	Fludioxonil	4.12	6.640	**248**, 249, 86
28	Myclobutanil	2.89	6.954	**179**, 245, 288
29	Trifloxystrobin	4.50	7.161	**172**, 116, 187
30	p,p-DDT	7.00	7.173	**235**, 165, 237
31	Iprodione	3.00	7.285	**314**, 184, 316
32	Bifenthrin	6.60	7.428	**181**, 165, 166
33	Bromopropylate	5.40	7.538	**341**, 339, 343
34	Fenazaquin	5.51	7.672	**145**, 160, 146
35	Pyridaben	6.37	7.985	**147**, 309, 207
36	Fenarimol	3.69	8.005	**139**, 251, 207
37	Etofenprox	6.80	8.413	**161**, 86, 135
38	Difenoconazole	4.40	8.752	**323**, 207, 281
39	Azoxystrobin	2.50	9.055	**344**, 207, 224
40	Famoxadone	4.65	9.233	**330**, 207, 224

K_ow_, partition coefficients octanol–water at 20 °C [32]; The quantification ions are in bold.

**Table 2:** The list of the investigated tinctures.

Type of Tinctures	Alcohol Content (%)	Description
Carmelite drops	40	10% extract from a mixture of herbs (lemon balm leaves, lemon perch, nutmeg, cinnamon bark, and clove flower) in 40% alcohol
Milk thistle drops	40	10% extract of milk thistle fruit in 40% alcohol
Willow flower drops	40	10% extract from the stem of a small-flowered willow in 40% alcohol
Echinacea drops	24.5	The drops contain the juice from the flowers of purple coneflower (*Echinacea purpurea*)
Grapefruit drops	66	Extract of grapefruit seeds and peel
Capsella drops	40	10% coconut stem extract in 40% alcohol

**Table 3:** The calculated MFs for pesticides in different types of medicinal drops. (Note: Pesticides are listed according to increased retention time).

Pesticides	MF/% Echinacea	MF/% Grapefruit	MF/% Carmelite	MF/% Capsella	MF/% Willow	MF/% Milk Thistle
Propham	3	23	35	8	8	74
o-Phenylphenol	45	75	−10	−46	26	60
Diphenylamine	236	106	85	124	111	109
Chlorpropham	409	97	134	69	89	125
Trifluralin	21	29	67	46	30	61
Hexachlorobenzene	11	14	−40	9	8	36
Diazinone	233	84	125	52	33	115
Tercbuthylazine	−38	65	−88	−56	−26	13
Lindane	1012	50	274	614	103	438
Pirimicarb	99	77	131	56	39	30
Dimethachlor	78	43	77	45	25	44
Acetochlor	199	39	80	47	34	35
Vinclozoline	70	69	62	44	41	37
Alalchlor	75	36	88	36	19	30
Tolclophos methyl	113	105	106	87	74	83
Pirmiphos methyl	129	110	131	108	74	71
Malathion	320	326	263	435	237	214
Chlorpyriphos	115	141	158	96	58	63
Triadimefon	269	117	−10	−22	349	−3
Cyprodinyl	1138	253	98	96	645	599
Penconazole	371	814	155	123	307	245
Tolylfluanid	201	198	170	144	163	119
Procymidone	104	113	52	57	62	40
Triflumizol	513	500	2379	1002	597	51
Bromophos ethane	129	179	94	105	100	45
Fludioxonyl	−42	−25	32	52	−15	−26
Myclobutanyl	635	234	126	325	115	50
Trifloxystrobin	1350	1540	322	532	437	689
p,p-DDT	181	182	59	65	144	139
Bifenthrin	−12	277	135	93	9	2
Bhromopropylate	271	527	358	420	253	129
Fenazaquin	637	1814	649	1329	1004	634
Fenarimol	135	142	98	235	88	87
Pyridaben	365	850	656	379	457	357
Etophenprox	532	616	687	432	521	357
Azoxystrobine	718	1772	1795	1192	1259	768
Famoxadone	68	49	86	98	56	60

**Table 4:** Validation parameters of the DLLME-GC-MS method for Carmelite drops.

Pesticides	R^2^	LOD µg/L	LOQ µg/L	LCL µg/L
Propham	0.9941	0.082	0.271	0.5
o-Phenylphenol	0.9736	0.009	0.030	0.05
Diphenylamine	0.9845	0.057	0.188	0.5
Chlorpropham	0.9984	0.225	0.743	1
Trifluralin	0.9750	0.002	0.007	0.01
Hexachlorobenzene	0.9764	0.011	0.036	0.05
Diazinone	09872	0.003	0.010	0.01
Tercbuthylazine	0.9725	0.111	0.366	0.5
Lindane	0.9799	0.416	1.373	5
Pirimicarb	0.9864	0.250	0.825	1
Dimethachlor	0.9882	0.028	0.092	0.1
Acetochlor	0.9875	0.123	0.406	0.5
Vinclozoline	0.9849	0.078	0.257	0.5
Alachlor	0.9997	0.094	0.310	0.5
Tolclofos-methyl	0.9947	0.018	0.059	0.1
Pirimiphos-methyl	0.9926	0.054	0.178	0.5
Malathion	0.9871	0.054	0.178	0.5
Chlorpyriphos	0.9778	0.005	0.017	0.05
Triadimefon	0.9890	0.039	0.129	0.5
Cyprodinil	0.9760	0.159	0.525	1
Penconazole	0.9877	0.011	0.036	0.05
Tolylfluanid	0.9947	0.089	0.294	0.5
Procymidone	0.9983	0.022	0.073	0.1
Triflumizol	0.9967	0.106	0.350	0.5
Bromophos-ethyl	0.9774	0.002	0.007	0.01
Fludioxonyl	0.9973	0.055	0.182	0.5
Myclobutanil	0.9764	0.040	0.132	0.5
Trifloxystrobin	0.9735	0.277	0.914	1
p,p-DDT	0.9836	0.098	0.323	0.5
Bifenthrin	0.9938	0.050	0.165	0.5
Bromopropylate	0.9741	0.011	0.036	0.05
Fenazaquin	0.9966	0.014	0.046	0.05
Fenarimol	0.9976	0.127	0.419	0.5
Pyridaben	0.9837	0.137	0.452	0.5
Etofenprox	0.9866	0.007	0.023	0.05
Azoxystrobine	0.9755	0.063	0.208	0.5
Famoxadone	0.9748	0.057	0.188	0.5

Note: LOD, limit of detection; LOQ, limit of quantification; LCL, lowest calibration level.

**Table 5:** Results of the analysis of real samples.

Type of Tinctures	Alcohol Content (%)	Concentration of Pesticides	RSD% *
Carmelite drops	40	o-Phenylphenol 16.47 µg/L Bromopropylate 0.67 µg/L	2 5
Echinacea drops	24.5	Tolclofos-methyl 2.24 µg/L	3

* Three repetitions.

## Data Availability

The original contributions presented in the study are included in the article, further inquiries can be directed to the corresponding author.
